# *Malassezia*: Zoonotic Implications, Parallels and Differences in Colonization and Disease in Humans and Animals

**DOI:** 10.3390/jof8070708

**Published:** 2022-07-04

**Authors:** Stefan Hobi, Claudia Cafarchia, Valentina Romano, Vanessa R. Barrs

**Affiliations:** 1 Department of Veterinary Clinical Sciences, Jockey Club College of Veterinary Medicine and Life Sciences, City University, Tat Chee Avenue, Kowloon, Hong Kong, China; 2 Department of Veterinary Medicine, University of Bari, Str. prov. per Casamassima Km 3, Valenzano, (Bari), 70010, Italy; claudia.cafarchia@uniba.it (C.C.); valentina.romano@uniba.it (V.R.); 3 Centre for Animal Health and Welfare, City University of Hong Kong, Kowloon Tong, Hong Kong, China

**Keywords:** dermatology, zoonotic diseases, fungi, *Malassezia*, yeasts, resistance, treatment, transmission, animals, humans

## Abstract

*Malassezia* spp. are commensals of the skin, oral/sinonasal cavity, lower respiratory and gastrointestinal tract. Eighteen species have been recovered from humans, other mammals and birds. They can also be isolated from diverse environments, suggesting an evolutionary trajectory of adaption from an ecological niche in plants and soil to the mucocutaneous ecosystem of warm-blooded vertebrates. In humans, dogs and cats, *Malassezia*-associated dermatological conditions share some commonalities. Otomycosis is common in companion animals but is rare in humans. Systemic infections, which are increasingly reported in humans, have yet to be recognized in animals. *Malassezia* species have also been identified as pathogenetic contributors to some chronic human diseases. While *Malassezia* species are host-adapted, some species are zoophilic and can cause fungemia, with outbreaks in neonatal intensive care wards associated with temporary colonization of healthcare worker’s hands from contact with their pets. Although standardization is lacking, susceptibility testing is usually performed using a modified broth microdilution method. Antifungal susceptibility can vary depending on *Malassezia* species, body location, infection type, disease duration, presence of co-morbidities and immunosuppression. Antifungal resistance mechanisms include biofilm formation, mutations or overexpression of *ERG11*, overexpression of efflux pumps and gene rearrangements or overexpression in chromosome 4.

## 1. Introduction

*Malassezia* are small thick-walled ovoid, ellipsoid or cylindrical commensal yeasts of warm-blooded vertebrates. Their genome of approximately 10 Mb is almost half the size of *Cryptococcus*, another basidiomycete of medical and veterinary importance [1,2]. The mycelial phase of *Malassezia* spp. has been observed naturally in some skin lesions and induced in specialized culture media incubated at 30 °C [2,3,4,5,6]. *Malassezia* species reproduce asexually by unipolar broad-based budding. The sexual form has not been detected, although the mating-type locus region has been identified [7]. 

An important characteristic of all *Malassezia* is their dependence on lipids for growth due to an absent fatty-acid synthetase gene and consequent inability to synthesize long-chain fatty acids. Although one species, *M. pachydermatis*, can readily grow on Sabauraud’s dextrose agar (SDA), a medium without lipid supplementation, it is still lipid dependent and its growth in this medium is due to the use of lipid fractions within the peptone, a component of SDA [2,8,9,10,11]. 

*M. furfur* was first identified on human skin in 1846 [12], but recently the genus has received more attention, not only because of its association with dermatological diseases in animals (dermatitis, otitis externa) and humans (pityriasis versicolor, atopic dermatitis, *Malassezia* folliculitis, seborrheic dermatitis) [2,13,14], but also due to its increased detection in systemic infections, especially in neonates and immunocompromised patients [15,16,17,18].

*M. pachydermatis*, originally thought to be part of the mycobiome in dogs and cats only, has now also been isolated from humans, production animals and from multiple exotic and wildlife species such as the sea lion, scarlet macaw, brown bear, American black bear, Eurasian badger, big anteater, common wombat, Mangaliza pig, wide-mouthed rhinoceros, Indian elephant, red fox, porcupine and coyote [19,20,21,22]. 

In this review, we use the One-Health paradigm to explore similarities and differences regarding carriage of *Malassezia* species in humans and companion animals, antifungal susceptibility, resistance mechanisms, *Malassezia*-associated diseases and treatment. The available evidence for transmission between animals and humans, directionality of transmission, and clinical relevance are also discussed.

## 2. Classification of *Malassezia* Yeasts

*Malassezia* yeasts belong to the family *Malasseziaceae*, order *Malasseziales* and class *Malasseziomycetes*. They are included in the morphologically highly diverse subdivision of *Ustilaginomycotina*, and due to their filament (hyphae) and reproduction characteristics, they are contained in the division of *Basidiomycota* [23,24,25,26]. 

Thus far, 18 *Malassezia* species have been identified from a variety of mammalian hosts and birds (Table 1) and further expansion of the genus is likely [27]. For species differentiation, locus analysis of specific ribosomal gene sequences, such as ITS, D1/D2, ß-tubulin, chitin synthetase 2 and large subunit polymerase 2, is used. For phylogenetic stem evaluation and species delimitation, whole genome sequencing (WGS) is necessary [1,27,28,29,30,31,32,33,34,35].

Recently, after the WGS of 28 representative isolates from 15 *Malassezia* species, concatenated protein sequences of 254 conserved orthologues were included in a phylogenetic analysis to resolve the taxonomy of the genus [27]. Similar to previous analyses [1,26], all species fell into three distinct clades [27] (Table 1).

## 3. *Malassezia* Species in the Environment and Possible Vectors

Although first isolated from the skin of humans, followed by other warm-blooded vertebrates, recent data have shown that *Malassezia* species have a much broader spectrum of ecological diversity than originally thought [36,37,38]. These yeasts have now been isolated from a range of environments, including marine water, anoxic oceans, hydrothermal vents, deep-sea to high arctic marine sediment and Antarctic soil [36,39,40,41,42,43,44,45,46,47,48,49,50,51,52]. *Malassezia* species also dominate the mycobiome of marine invertebrates, such as sponges and corals, and have been identified in healthy and diseased marine algae [36,53,54]. In addition, *Malassezia* species have been isolated from soil nematodes, cone-snails, olive fruit-flies and orchid roots [55,56,57,58]. A potential role for nematodes and flies as vectors for *Malassezia* has been speculated [55,58,59,60].

It is now apparent that *Malassezia* species are among the most widespread fungi on Earth [36,37,38]. Their evolutionary trajectory involves adaptation from an ecological niche in plants and soil to the mucocutaneous ecosystem of animals [36,37,38]. This has been facilitated by the loss of complex carbohydrate metabolism genes (glycosyl hydrolase encoding) and a genus-wide gain of lipid hydrolases including lipases, phospholipases and acid sphingomyelinases that are required to degrade and use skin- or mucosa-associated lipids [1,36,37,38].

## 4. *Malassezia* Species and Their Role as Commensals in Humans

Twelve *Malassezia* species have been isolated from human skin [6,16,34,61,62,63,64,65,66,67,68,69,70,71,72,73,74,75] (Table 1). *Malassezia arunalokei* is the only species isolated from humans that has not been isolated from animals, with the exception of dogs [74,76].

*Malassezia* species colonization of the skin starts directly after birth, increases until around 12 months of age, and then remains relatively static until puberty, when another significant quantitative increase in colonization occurs, associated with increased sebaceous gland activity and changes in the lipid composition of the skin [6,77]. After puberty, *Malassezia* species comprise 50 to 80% of the human mycobiome [78,79,80,100]. The limited data currently available about cutaneous mycobiomes in preterm and term neonates shows that *Malassezia* species distribution on the skin of neonates and children varies between studies, but *M. globosa*, *M. furfur*, *M. sympodialis* and *M. restricta* are the most prevalent species described [14].

In contrast, *M. restricta* and *M. globosa* dominate the mycobiome of both healthy and diseased skin in adult humans, followed by *M. sympodialis*, albeit at a much lower frequency than the former two [1,62,69,81,82,83,84,85,86,87,88,89,90,91]. *M. furfur* can be common at certain body sites (e.g., toe-web space) in healthy individuals but is not a dominating species overall. Instead, this species is more frequently isolated from skin diseases, such as psoriasis vulgaris and pityriasis versicolor [1,5,73,89,90,91,92,93]. 

**Figure 1:**
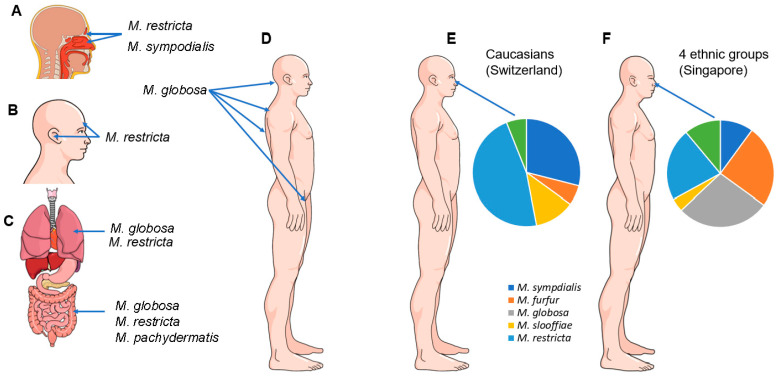
Predominant *Malassezia* species found as commensals in the sinonasal cavity (**A**), on the skin of the forehead and external ear canal (**B**), in the lungs and gastrointestinal tract (**C**) and on the skin of the occiput, back and groin (**D**). The relative diversity of *Malassezia* species found in healthy skin is shown in (**E**,**F**). The predominant species isolated from the culture of the skin on the side of the nose of Caucasians in Switzerland were *M. restricta* and *M. sympodialis,* while *M. globosa* was absent. In contrast, sampling from the skin of the noses of 4 ethnic groups (Chinese, Malay, Indian and Caucasian) in Singapore overall revealed *M. globosa*, *M. furfur* and *M. restricta* to be the dominant species [95].

Climate and ethnicity also impact the carriage of *Malassezia* species [94,95]. In a study by Leong et al. in 2019, people in Singapore of four different ethnicities (Chinese, Malay, Indian and Caucasian) carried a higher number of *Malassezia* species and showed greater species diversity and evenness than Caucasians in Switzerland. The predominant species (isolated by culture from the skin of the side of the nose) in the latter were *M. restricta* and *M. sympodialis*, while *M. globosa* was absent. In contrast, sampling from the same site in the four ethnic groups in Singapore showed *M. globosa*, *M. furfur* and *M. restricta* were the dominant species. Caucasians from the two locations showed different species distributions, with *M. restricta* being twice as common among those in Singapore, while *M. globosa* was absent in Swiss Caucasians. The same study associated the lower temperature and humidity of Switzerland compared to Singapore with a lower positive culture rate and lower species diversity [95] (Figure 1). From other studies, it can be concluded that *M. restricta* plays a dominant role as a skin commensal in Europe, whereas *M. globosa* comparatively dominates in Asia [13,96].

Several studies have shown that sex and body site also influence the species of *Malassezia* species present on the skin and their abundance [1,88,91,97,98,99,100]. Site-specific species include *M. restricta*, which favors colonization of the external ear canals, retroauricular crease and forehead and *M. globosa*, which is most commonly isolated from the back, occiput and groin [100,101]. 

A Japanese study in 2010 quantified *Malassezia* colonization of the skin of the cheek using real-time PCR and determined associations with gender and age in 770 healthy individuals [99]. Total *Malassezia* DNA in males stayed constant from age 0 until around 9 years of age, with a progressive increase each year thereafter until the age of 16 to 18. In females, total *Malassezia* DNA increased until the age of 12, decreased between the ages of 19 and 22, and then increased again between the ages of 30 and 39. In both genders, there was a gradual decrease in *Malassezia* species abundance over the course of life. Overall, males tended to have more abundant *Malassezia* DNA than females, and *M. globosa* and *M. restricta* were the dominant species for both for all ages. 

*Malassezia* species carriage at different skin locations was investigated using culture-based methods. No significant differences between the genders were found. While *M. restricta* dominated the scalp and *M. sympodialis* dominated the trunk, *M. globosa* was about equally common at both locations [102]. 

Other factors that may influence the colonization of *Malassezia* species include host factors (immune response, body secretion, skin occlusion), other skin inhabitants (e.g. parasites, other microbes) and environmental parameters, including exposure to ultraviolet light [96]. Even the birth process itself has a significant impact. If a baby is born via natural delivery, its skin microbiota resembles the mother’s vaginal communities, but if delivered via caesarian section, it represents the mother’s skin surface population [103,104,105,106,107]. In addition, vaginal birth is associated with a higher abundance of *Malassezia* [108,109]. 

*Malassezia* species were previously thought to be commensals of the skin only. Although the skin is the primary ecological niche, more recent data demonstrate that these yeasts also colonize the mucosa of the sinonasal and oral cavities, as well as the gastrointestinal and lower respiratory tract [110,111,112,113,114,115]. *Malassezia* species are dominant members of the mycobiome of the sinuses, with *M. restricta* and *M. sympodialis* most frequently detected [116]. *Malassezia* also comprise 30% of the gastrointestinal mycobiome, with three species detected—*M. globosa*, *M. restricta* and *M. pachydermatis* [117]. The fungal burden in the lungs of healthy people is relatively low. In one study, using a metagenomic approach, the lung mycobiome was characterized by a high proportion of basidiomycetes, including *M. restricta* and *M. globosa* [118], while in another ascomycetes, including *Candida* species, were most abundant [119] (Figure 1). 

## 5. *Malassezia* Species and Their Role as Commensals in Companion Animals

Using culture-based techniques, *Malassezia* species have been identified as the most common yeast colonizing healthy canine skin [120,121]. Metagenomic approaches reveal that, in contrast to humans, Ascomycota, especially *Alternaria* and *Cladosporium* species, are the most abundant fungal species on the skin of healthy dogs and cats [122,123].

Overall, eleven *Malassezia* species have been isolated from cats and seven from dogs [2,124,125,126,127,128,129,130,131,132,133,134,135,136,137,138,139,140,141,142] (Table 1). Culture-based studies clearly favor *M. pachydermatis* as the dominant species colonizing the skin of dogs and cats [124,125,126,128,135,143,144,145,146]. In one recent study using metagenomics and quantitative PCR (qPCR), *M. restricta* and *M. globose,* but not *M. pachydermatis*, were identified as the dominant species colonizing healthy feline skin [139]. 

**Figure 2:**
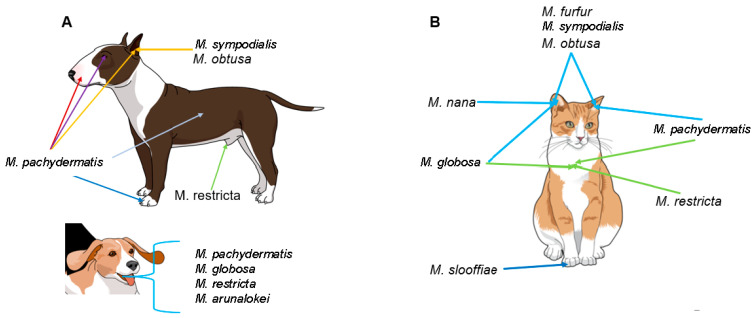
*Malassezia* species colonization in healthy dogs (**A**) and cats (**B**).

*Malassezia* abundance and species diversity are influenced by body site, genetic predispositions and concurrent diseases [2,135,136,137,140,141,146,147,148]. *M. pachydermatis* is more frequently isolated from dogs from perioral and interdigital skin than from the back or ventral body sites, such as the axillae or groin [2] (Figure 2).

In cats, the external ear canal is most commonly colonized by *M. pachydermatis,* followed by other species such as *M. furfur*, *M. globosa*, *M. sympodialis*, *M. obtusa* and *M. nana* [134,142,149,150,151,152]. *M. nana* is the most common skin and ear isolate after *M. pachydermatis,* with one specific genotype dominating [136,137]. Claw folds of cats are a particular niche for *M. slooffiae* [135,136,137] (Figure 2).

Two specific feline breeds, the Devon Rex and Sphynx, harbor high *Malassezia* species loads, with a dominance of *M. pachydermatis* [135,146,147]. Cats with otitis externa also have a higher abundance of *Malassezia* species in the ear canals compared to healthy individuals. The most prevalent *Malassezia* species were *M. pachydermatis* (57.7–62.62%), followed by *M. globosa* (11.4–22.2%), *M. furfur* (2.4–17.2%), *M. obtusa* (15.4%), *M. slooffiae* (7.3%), *M. sympodialis* (1–4.1%) and *M. restricta* (1.6%) [134,152].

In dogs, similar to cats, *M. pachydermatis* dominates the healthy ear canal, although other species, such as *M. sympodialis* and *M. obtusa,* can also be detected [142,149,150,153] (Figure 2). In diseased canine ears, the colonization rate increases, but *M. pachydermatis* remains most prevalent, followed by *M. sympodialis*, *M. furfur*, *M. obtusa*, *M. globosa* and *M. restricta*. This has mostly been shown by cultural and biochemical evaluation methods [142,150,153,154]. 

Allergic dogs have a higher abundance of *Malassezia* species, especially of *M. pachydermatis*, compared to healthy dogs [140,148] as well as a decreased overall diversity [155].

In particular, in dogs with atopic dermatitis, the isolation of *M. sympodialis* associated with *M. pachydermatis* and/or *M. furfur* has also been reported [156]. The coexistence of *M. pachydermatis* with other *Malassezia* species makes the pathogenic role determination of a single *Malassezia* species challenging.

Other body sites where *Malassezia* species are commensals have not been well characterized in dogs and cats. Low numbers of *M. pachydermatis* are present on the corneal surfaces of healthy dogs [120]. 

In a recent study, the mycobiome of the healthy canine oral cavity was found to be dominated by *Malassezia* species and *Cladosporium* species. *Malassezia arunalokei*, *M. restricta*, *M. pachydermatis* and *M. globosa* have all been detected in the oral cavity of healthy and diseased dogs (Figure 2), although no correlation was found between these *Malassezia* species and periodontal disease [76]. 

In the gastrointestinal tract of dogs, Ascomycota and Basidiomycota are the most numerous, with *Candida* as a major genus [157,158,159,160]. *Malassezia* species have not yet been detected. In cats, even less data are available, but Ascomycota seem to be dominant [158].

The urinary mycobiome of healthy dogs was recently characterized, and reads of several fungal genera in very low abundance were detected, including *M. restricta* [161].

## 6. *Malassezia* Species in Other Animals

*Malassezia* species have been isolated from multiple mammalian and avian species (Table 1). In pigs, *Malassezia* species, especially *M. sympodialis* and *M. slooffiae,* were isolated from 73% of healthy ear samples but not from multiple skin sites [162]. *M. pachydermatis* has been isolated from pigs with otitis externa and from the skin of healthy farmed pigs [163,164]. One study [165] compared *Malassezia* species detection rates and species from healthy porcine ears among different porcine breed and age groups, showing that, similar to humans and companion animals, genetic factors and age can impact *Malassezia* abundance and diversity [2,85,95,141,145,152,166]. Overall, *Malassezia* were isolated from 22.5% of sampled pigs, including *M. pachydermatis*, *M. furfur* and *M. sympodialis*. *M. pachydermatis* was found in all breeds but not in adults of large breeds, whereas *M. furfur* and *M. sympodialis* were only present in adult pigs of large breeds [165].

Several *Malassezia* species were isolated from multiple skin sites of 12 to 60% of healthy cattle using culture-based techniques [149,167] (Table 1). One study showed a clear difference in the species isolated in cases of otitis externa depending on the time of the year, with thermotolerant *M. sympodialis* dominating in summer and less thermotolerant *M. globosa* being predominant in winter [168].

A variety of *Malassezia* species have been isolated from the skin of horses, sheep and goats (Table 1). In goats, skin infections have been associated with *M. pachydermatis* and *M. slooffiae* [169,170].

Among rabbits, *M. cuniculi* is commonly detected in healthy skin and ears [30,171]. In one study, rabbits bred for meat consumption were more frequently colonized with *Malassezia* species compared to pet rabbits [171]. In contrast to humans, *Malassezia* species were more commonly present in young rabbits (<3 months of age), and diet impacted *Malassezia* species prevalence. 

Among different bird species, *Malassezia* species have been isolated from healthy and diseased sites, including beak (*M. brasiliensis*, *M. psittaci*), feathers and wings (*M. pachydermatis*, *M. furfur*), oropharynx (*M. pachydermatis*, *M. furfur*, *M. brasiliensis*, *M. psittaci*), and feces (*M. pachydermatis*, *M. furfur*) [20,125,172]. *M. sympodialis* has also been commonly found in diseased combs of adult chickens [173].

## 7. Zoonotic and Reverse Zoonotic Transmission of *Malassezia* Species

There is now ample evidence that different *Malassezia* species are shared between humans and animals (Table 1). However, some genotypes within a species might be host adapted or linked to a particular host site location or skin disorder [27,30,31,74,110,174]. In particular, sequence analyses of the LSU rDNA showed distinct *Malassezia* species subtypes on different host species [110]. Sequence analysis of IGS1 distinguished specific *M. globosa*, *M. restricta*, and *M. pachydermatis* variants in seborrheic dermatitis and atopic eczema and on the healthy skin of humans and animals [85,174]. Among *M. pachydermatis,* eight IGS1 subtypes were identified and subtype 3D was mainly associated with skin lesions [175]. Additionally, *M. pachydermatis,* frequently isolated from cats and dogs [176,177,178,179,180], but rarely from human skin [62,181,182] was known to cause fungemia in people, especially in neonates [34,63,183,184,185,186,187,188,189,190,191]. However, newborn babies have skin colonization by *M. sympodialis* and *M. globosa,* but not by *M. pachydermatis* [78,79,80,100]. Thus, the ease with which these yeasts can be transmitted from one body site to another [192] or between animals and their owners [182] makes us hypothesize that zoonotic and reverse zoonotic transmission of these yeast species can occur. 

In particular, the carriage of *M. pachydermatis* in healthy and diseased dogs with allergic dermatitis or otitis externa was compared to healthy human owners [182]. *M. pachydermatis* DNA was identified on the palms of over 90% of pet owners, regardless of the disease state of their dogs. Based on culture results indicating the relative abundance of *Malassezia* species, owners of affected dogs were 11 times more likely to be culture positive than owners of healthy dogs [182].

The zoophilic potential of *M. pachydermatis* was first postulated by Dr. Gueho [193] but was clearly confirmed ten years later when an outbreak of neonatal fungemia caused by *M. pachydermatis* was investigated [184]. The strain implicated in the outbreak was isolated from a health care worker’s hands, from contaminated equipment and from dogs belonging to three health care workers working in the involved intensive care nursery unit. One or several healthcare workers likely contaminated the nursery environment and their patients after transient colonization of their hands by the organism. After optimizing hand hygiene, no further cases were reported and all cultures from staff members tested negative [184]. 

Other studies have also demonstrated that hospitalized infants can be colonized by *Malassezia* species, especially *M. pachydermatis* and *M. furfur,* via contact with their parents or healthcare workers or indirectly via incubator surfaces [16,77,183,184,194,195,196]. Healthcare workers can then further transmit the organism from one infant to another via their hands. Through this mechanism, several *Malassezia* species outbreaks have occurred in the past [184,197,198].

Carriage of *M. pachydermatis* in humans was detected in low numbers on the scalp and palms of 12% of healthy individuals in one study [181], and on the skin of 5% of healthy medical students in another [62]. In other studies it was not detected at all in healthy individuals, and overall appears to be a rare, transient colonizer of human skin [6,102]. Similarly, other studies have found no causal associations between *M. pachydermatis* and human *Malassezia*-associated skin conditions, including seborrheic dermatitis and pityriasis versicolor [62,199]. 

While there is evidence that *M. pachydermatis* can be transmitted between dogs and humans, further investigations into the genotypes involved, and the strain characteristics are warranted [184,188,189,190,200]. The relatively recent discovery of *M. pachydermatis* as a commensal of the human gut introduces another potential reservoir of infection in humans by this species [117].

There is phenotypic and phylogenetic evidence that species with high host diversity, such as *M. furfur*, are undergoing diversification to enable successful adaptation to different hosts [201]. Strains from different animal species remain closely genetically related, but the extent and frequency of zoonotic or reverse zoonotic transmission have not been investigated. 

## 8. Superficial *Malassezia*-Associated Diseases in Humans and Animals

### 8.1. Malassezia-Associated Dermatological Diseases in Humans

**Figure 3:**
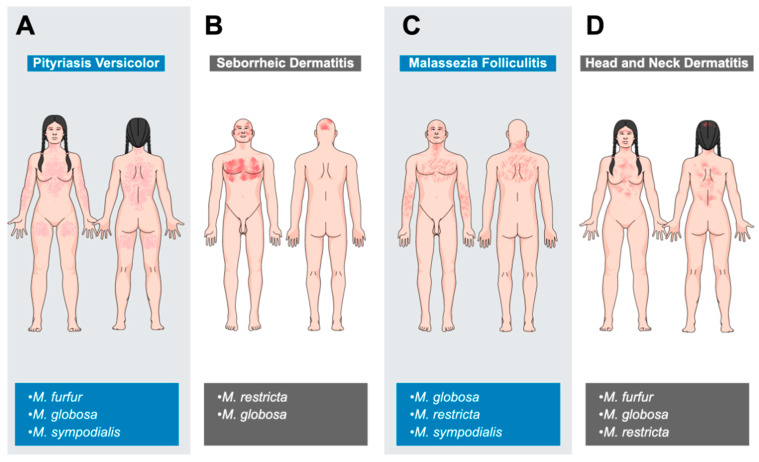
Overview of *Malassezia*-associated skin diseases in humans. Typical affected areas for pityriasis versicolor (**A**), seborrheic dermatitis (**B**), *Malassezia*-associated folliculitis (**C**) and head and neck dermatitis (**D)**, as well as commonly involved *Malassezia* species, are shown.

The most common *Malassezia*-associated skin diseases in human patients are pityriasis versicolor, seborrheic dermatitis, *Malassezia* folliculitis and atopic eczema [6,13,38,202,203,204]. The skin sites and species involved in these diseases are shown in Figure 3.

#### 8.1.1. Pityriasis Versicolor

Pityriasis versicolor, sometimes called tinea versicolor, is a common disease worldwide, with a prevalence of up to 50% in hot and humid regions. There is no gender or ethnic predisposition [205,206]. The disease is most commonly seen in young adult to adult patients, correlating with increased sebaceous gland activity and altered lipid composition of the skin around this time. The disease is clearly associated with *Malassezia* species, especially *M. furfur*, *M. globosa* and *M. sympodialis*. A combination of factors including genetics, warm and humid environment, immunodeficiency, pregnancy, oily skin or application of oily topical substances lead to a transformation of resident *Malassezia* species into a pathogenic filamentous form. Most patients have multiple affected areas characterized by well-demarcated, oval, hyper- or hypopigmented macules with a fine scaly surface. These lesions are variably pruritic and the neck, trunk and proximal extremities are commonly involved [207,208,209] (Figure 3). Diagnosis is usually made clinically, but in individual cases, Wood’s lamp examination (coppery-orange fluorescence) or microscopic examination of fungal elements may be needed [6,210,211]. 

#### 8.1.2. Seborrheic Dermatitis

Seborrheic dermatitis also occurs worldwide, with ‘normal’ and dandruff forms affecting around 5% and up to 50% of the population, respectively. There is also an HIV-associated form. There is no ethnic predisposition, but males are clearly predisposed. Disease is mainly seen in infants and adults [212,213,214]. The etiology is not completely clear but involves an interplay of skin flora, lipid composition on the skin surface, skin barrier integrity, immune response to *Malassezia* species and individual host factors. Increased sebaceous gland activity, immunodeficiency, neurological and psychological diseases, certain drugs and environmental factors such as low humidity and temperature are risk factors for seborrheic dermatitis [213,215,216]. *M. restricta* or *M. globosa* are typically isolated from active lesions and antifungal treatment usually leads to significant clinical improvement. Other species can be isolated, including *M. furfur*, *M. sympodialis*, *M. obtusa* and *M. slooffiae* [38,217,218,219]. The scalp, face and chest are most commonly affected, although in infants, the diaper area, neck and axillae may also be involved (Figure 3). Skin lesions are often inflamed, pruritic and present at one or multiple locations. They include poorly defined follicular papules and plaques, fine white scales, and yellow crusts. In the mild dandruff form, no inflammation but a fine, mild scaling on the scalp and beard dominates [220,221]. 

#### 8.1.3. *Malassezia* Folliculitis

*Malassezia* folliculitis is another common worldwide disease with a prevalence of 1 to 17%. It occurs more commonly in young to middle-aged adult males [222,223,224]. Follicular occlusion or a disturbance of the normal cutaneous flora leads to an abnormal proliferation of *Malassezia* species and the development of disease. Common associated species include *M. globosa*, *M. restricta* and *M. sympodialis* [6,202,224,225,226,227,228]. Predisposing factors include hot and humid climate, excessive sweating, non-breathable clothing, application of make-up or sunscreens, certain drugs (antibiotics, glucocorticoids) and immunosuppression [6,224,229,230]. The disease typically involves the face, upper back, extensor surfaces of the arms, chest and neck (Figure 3). In almost 75% of cases, more than one location is affected. Lesions include small but pruritic follicular papules and pustules. This presentation is often mistaken for acne or bacterial folliculitis [223,224,231,232]. 

#### 8.1.4. Atopic Dermatitis (Head and Neck Dermatitis)

Atopic dermatitis (AD) is a common, chronic, inflammatory and pruritic disease, affecting 10 to 25% of children and 1 to 2% of adults. Head and neck dermatitis (HND), a subtype of AD, mostly occurs in adolescence and adulthood in individuals with a history of IgE-mediated AD. There is no gender or ethnic predisposition [233,234,235,236]. The etiology is incompletely understood, but it is clear that *Malassezia* species play an important role in disease pathogenesis. The high activity of sebaceous glands at affected sites, together with the skin barrier disruption of the atopic disease, allow *Malassezia* species to proliferate, leading to increased exposure to the immune system, triggering a humoral and cell-mediated immune response [237,238,239,240,241,242,243]. Some involved *Malassezia* antigens have been well characterized (*M. globosa*—MGL_1304; *M. sympodialis*—Mala s 8; *M. restricta*—Mala r 8) and have been identified in the sweat of patients, leading to aggravated clinical signs, especially after intense sweating [244,245]. These antigens have also shown variable histamine-releasing properties [246]. *Malassezia* species isolated from disease-associated sites have included *M. furfur*, *M. obtusa*, *M. globosa*, *M. restricta* and *M. sympodialis,* but there was no significant difference in isolation compared to healthy individuals [240,247]. Others found a higher colonization rate by *M. furfur,* as well as a lower colonization rate by *M. globosa* and *M. sympodialis,* in affected AD patients [62]. Specific genotypes of *M. globosa* and *M. restricta* have also been identified as colonizing AD skin [65,174]. 

HND patients have erythema and erythematous plaques on the forehead, eyelids, perioral, neck and upper trunk together with variable pruritus (Figure 3). In severe cases, the whole face may be involved, leading to the term “red face”. With the chronicity of the disease, lichenification and scaling can occur [245,248]. Wheal-like, edematous changes have also been described [245]. 

### 8.2. Malassezia Dermatitis and Otitis Externa in Animals

In dogs and cats, *Malassezia* dermatitis and otitis externa are commonly encountered in daily practice [141,156,249] but they can also be seen in farm animals, especially horses and goats. The prevalence of *Malassezia*-associated skin diseases in farm animals may be underestimated [169,170,250,251,252,253,254,255,256]. *Malassezia* dermatitis and otitis externa have also been reported in many other animals, including sea lions, fennecs, okapi, dromedaries, rhinoceros, canaries and pinnipeds [21,163,257,258,259,260,261,262]. 

Concurrent *Malassezia* dermatitis and sarcoptic or demodectic mange are occasionally seen in lagomorphs or hamsters, respectively [263,264,265]. Some specific dog and cat breeds have a higher risk of *Malassezia* dermatitis [135,145,266,267,268,269] (Table 2).

In veterinary *Malassezia*-associated dermatitis, typical cutaneous manifestations include alopecia, erythema, scaling, crusts and accumulation of greasy, malodorous, brown to black keratosebaceous debris. In chronic infections, lichenification and hyperpigmentation may also be present. The intensity of pruritus is variable [2,141,179,254,255,256,260,265,266,270,271]. 

**Figure 4:**
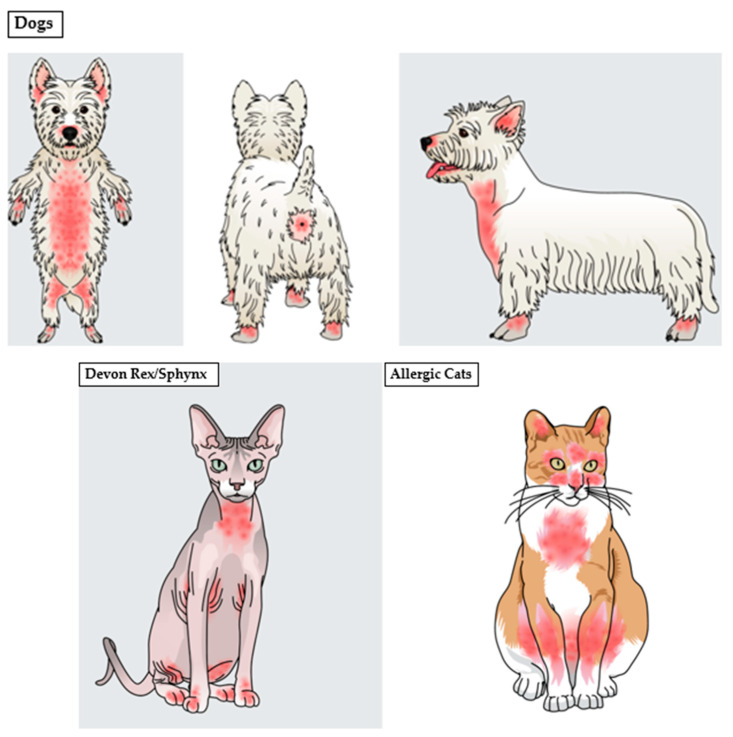
Common affected skin sites in dogs and cats with *Malassezia*-associated dermatitis and otitis externa. In cats, generalized diseases can also be seen.

In canine patients, an infection or overgrowth with *Malassezia* species is most commonly associated with allergic diseases (flea bite hypersensitivity, food allergy, atopic dermatitis), ectoparasitic infestations, superficial pyoderma, occasionally with endocrinopathies (hypothyroidism, hyperadrenocorticism, diabetes mellitus), keratinization disorders and rarely with autoimmune diseases [272,273,274,275]. Common involved areas include the external ear canal, pinnae, lips, muzzle, ventral neck, ventral body sites, medial hind limbs, perianal site and paws [270,276] (Figure 4).

In dogs with environmental allergies, the clinical signs of *Malassezia* dermatitis often mimic, or even worsen, those of atopic disease [270]. It has been shown that affected patients show elevated levels of *Malassezia*-specific IgG and IgE in their serum [277]. In addition, immediate hypersensitivity reactions were observed in canine atopic patients in which *M. pachydermatis* extracts were intradermally injected or after passive transfer of atopic serum to healthy recipient dogs using the Prausnitz–Küstner (P-K) technique [278,279]. Together with the frequent isolation and higher colonization rate of *Malassezia* species on and from the skin of these patients, their relevance and contribution, especially *M. pachydermatis*, to disease pathogenesis has been demonstrated [140]. Four major allergens of *M. pachydermatis* with a size of 45, 52, 56 and 63 kDa were detected in more than 50% of atopic dogs in a study by Chen et al. in 2002 [280].

While *Malassezia* dermatitis in cats can be associated with similar diseases in dogs, especially skin fold dermatitis and hypersensitivities [2,141,281,282] there are also more exclusive presentations, such as idiopathic facial dermatitis [141,270,283,284,285], acne [2,141,286,287], paraneoplastic alopecia [2,141,288,289,290,291,292,293,294], thymoma-associated exfoliative dermatitis [2,141,295,296], FIV-associated dermatitis [2,141,297], Feline leukemia virus or Feline immunodeficiency virus infection [298] and superficial necrolytic dermatitis [2,141,299]. In most cats, common affected body regions include the pinnae, face, chin, neck, limbs and abdomen, while in Devon Rex and Sphynx cats, the ventral neck, axillae, groins and paws dominate [2,145,166] (Figure 4). 

*Malassezia*-associated otitis externa in animals can be unilateral or bilateral and is associated with ear scratching, head shaking and brown to black, often malodorous discharge. The pinnae, especially near the orifice of the ear canal, are often also affected [2,141,252,300]. In a recent canine study, a painful, erosive to ulcerative form of otitis externa with a watery brown to black discharge caused by *Malassezia* species was described [301]. In contrast, otomycosis due to *Malassezia* spp. is considered rare in humans [302,303]. 

### 8.3. Miscellaneous Forms of Superficial Malassezia-Associated Diseases

Occasionally, *Malassezia* species can also infect the nails of humans and the claws of animals. In companion animals, paronychia with erythema, swelling and a waxy brown to black discharge is common, while in humans, subungual hyperkeratosis and onycholysis can be seen [85,141,304,305,306]. 

Another potential site of superficial *Malassezia* infection is the cornea. There are sparse reports of keratomycosis in humans and dogs associated with *M. furfur* and *M. restricta* in humans and *M. pachydermatis* in dogs [307,308,309]. Interestingly, one affected dog [309] and a human patient [308] both had diabetes mellitus and in all described cases immunomodulatory or antibiotic drugs were used. These predisposing factors could have facilitated *Malassezia* species overgrowth. The burden of corneal colonization by *Malassezia* species significantly increases in cases of corneal ulceration [120,310]. Whether *Malassezia* species could have a primary pathogenic role in some cases of corneal ulceration requires further investigation.

## 9. Systemic Infections and Chronic *Malassezia*-Associated Diseases in Humans and Animals

### 9.1. Fungemia and Systemic Infections

Of the 18 *Malassezia* species, only three are known to cause fungemia—*M. furfur*, *M. pachydermatis* and *M. sympodialis*. In the former two, one specific genotype is involved [14,198,311,312]. In fungemic patients, *M. furfur* is most frequently isolated, followed by *M. pachdermatis* and *M. sympodialis* [14,16,313]. 

Since the first report of systemic infection by an unspecified *Malassezia* species in 1979 [314], systemic infections have been described with increasing frequency [14,15,16,18,198], likely due to growing recognition of the pathogenic potential of *Malassezia* species, as well as improved detection methods [14,110]. 

The skin plays a significant role in the development of fungemia as both a reservoir of *Malassezia* species and a portal of entry into the bloodstream by *Malassezia* species when it is compromised [14]. Predisposing factors for fungemia include premature birth, hospitalization and duration of stay in a neonatal intensive care unit, immunosuppression, peritoneal dialysis, presence of central venous catheter, total parenteral nutrition with lipid supplementation (especially in neonates), invasive surgical procedures, long-term or broad-spectrum antimicrobial administration, chronic illnesses and topical application of soybean oil containing products [16,184,185,315]. Parenteral lipids are not only favorable for *Malassezia* species growth but can also reduce the immune response of a patient by the generation of reactive oxygen species, which decrease neutrophil phagocytosis [183,184,316,317].

The pathogenesis of *Malassezia* fungemia is not fully understood. Since only particular genotypes of *M. furfur* or *M. pachydermatis* are associated with fungemia, pathogen virulence factors are likely important determinants of systemic infection [198,311,312]. *Malassezia* species possess a number of virulence factors, including lipases, phospholipases, metabolites (indirubin, indole carbazole, pityriacitrin and others), nanovesicles, cell membrane µ-opioid receptors, hydrophobicity, adherence and the ability to form biofilm [38,318,319,320,321,322,323,324,325]. Of these, increased phospholipase activity and the release of allergen-enriched nanovesicles are often related to more severe disease and fungemia [312,318,321,322,326].

Pathogenic *Malassezia* strains associated with fungemia are either already present colonizing the patient’s skin or are transmitted to the skin through interactions with healthcare worker’s hands or contaminated medical devices, materials and/or parenteral solutions [14,16,77,183,184,194,196]. 

Severe illness, the administration of immunosuppressive, antifungal or broad-spectrum antimicrobial drugs or parenteral lipids, poor anatomic conformation and/or premature age lead to an impaired immune state. Different combinations of such factors enable invasion of the body at an entrance point, such as a surgical wound or an intravenous catheter site [14,38,194,327,328]. 

Hematogenous dissemination of *Malassezia* species can involve infection of the heart, lungs and, less commonly, the kidneys, pancreas, liver, spleen, brain and skin (multiple cutaneous pustules) [183,184,316,317]. Biofilm formation facilitates local replication and further shedding of the organism into the blood system [317,329,330,331]. 

Systemic infections with *Malassezia* species include a broad range of presentations, from single-organ infection to fungemia, and can be fatal. Single-site infections include meningitis [332,333], endocardial mass [334], pneumonia [335,336], peritonitis [314,337,338], osteomyelitis [339], septic arthritis [339], sinusitis [340] and mastitis [341]. 

Clinical signs of systemic *Malassezia* species infection in infants include fever, respiratory distress from pneumonia or bronchopneumonia, lethargy, bradycardia, seizures and cyanosis. Infected infants often show hepato- and splenomegaly. The main hematological findings are leukocytosis or leukopenia and thrombocytopenia [183,184,185,342,343,344,345]. 

Infections in children and adults are characterized by fever, chills, myalgia, nausea, vomiting and respiratory distress. Haematological findings include leukopenia (rarely leukocytosis) and thrombocytosis [329,346,347,348]. 

The diagnosis of *Malassezia*-associated fungemia is challenging due to its special needs for growth including lipid dependency. It is recommended to directly culture blood or central venous catheter tips on lipid-rich culture media via blood culture specimen tubes and not to use an automated blood culture system [14,313,349]. In addition, since human blood can have inhibitory and toxic effects on yeasts, the addition of 3% palmitic acid may favor positive detection [350].

Thus far, *Malassezia*-associated fungemia has not been reported in animals. 

### 9.2. Chronic Diseases in Humans and Animals

In patients with HIV infection, the burden of the *Malassezia* species in the gut and on the skin of individuals with seborrheic dermatitis is significantly increased, associated with low numbers of CD4+ helper cells/Th17 cells. This overgrowth of *Malassezia* species is a risk factor for fungemia and other *Malassezia*-associated infections, including HIV-associated seborrheic dermatitis [115,351,352,353]. 

In patients with inflammatory bowel disease (IBD), including Crohn’s disease, *Malassezia* species dominate the gastrointestinal mycobiome [354,355,356]. *M. restricta* colonization, especially in the sigmoid colon, can increase disease severity by intensifying the inflammatory response [356]. This effect is strongly associated with the presence of the Crohn’s disease risk allele altered caspase recruitment domain 9 (CARD9 S12N). CARD9 is an adapter protein of the CARD-CC family that mediates pattern recognition signaling and is essential for fungal defense [115,354,355,356,357,358,359]. In mice models, the same authors showed the capability of *M. restricta* to cause significant changes to the colon, including colon shortening, mucosal erosion and crypt destruction [356]. 

It has been speculated that *Malassezia* species could have a pathogenic role in the development or progression of colorectal cancer since affected individuals have gastrointestinal mycobiome dysbiosis with a higher burden of *Malassezia* species compared to healthy individuals [115,360,361,362]. However, whether this is an effect or a cause of cancer remains to be proven. *Malassezia* species have been found to play a causal role in pancreatic ductal adenocarcinoma (PDA) associated with migration from the gut to the pancreas [361]. In human and murine PDA, cancerous pancreatic tissue contained a 3000-fold higher burden of fungi compared to healthy pancreatic tissue and was specifically enriched for the *Malassezia* species. The oncogenic pathway was also identified as the activation of mannose-binding lectin, which drives the complement cascade and promotes oncogenesis [361].

A role for *Malassezia* species in neurodegenerative diseases, such as Alzheimer’s disease and Parkinson’s disease, has been speculated due to their frequent detection in affected areas of brain tissue [363,364,365,366]. The source of *Malassezia* species is fungemia due to breaches of the cutaneous or gastrointestinal barriers. However, whether their presence reflects opportunistic colonization of damaged tissue or is causal has not been determined. Similar studies in veterinary medicine are lacking. 

## 10. Antifungal Susceptibility Testing

### 10.1. Methodology

Usually, established testing concentrations are used as a reference for systemically applied drugs at their recommended doses [367,368]. Topically, much higher concentrations can be reached, important for topical therapy and thereby susceptibility testing methods would need to be adjusted [369,370,371]. Since *Malassezia* species are involved in common diseases and can potentially cause deep infections, fungemia or even death, susceptibility testing becomes a necessary and very important tool [14,15,16,18,110]. Even if there are standard proposed guidelines for testing the susceptibility profile of filamentous fungi and yeasts, it is difficult to implement these methods with *Malassezia* species due to their special needs and growth characteristics [372,373]. As a consequence, and due to the lack of standardization, different procedures were proposed with culture medium, inoculum size, incubation time, and criteria used to determine MIC endpoints largely vary among the studies, thus making it difficult to interpret the data in the literature [374]. The susceptibility of *Malassezia* species to antifungal compounds has been tested using different methods, including a modified Clinical and Laboratory Standards Institute (CLSI) broth microdilution protocol [375,376,377,378] and agar-based diffusion methods (Disk Diffusion – DD and the E test—ET) [379,380,381,382,383]. However, the agreement analysis between agar-based diffusion methods and modified CLSI standard reference procedures still needs to be better investigated. Overall, DD might not represent a valid alternative for determining the susceptibility of *Malassezia* yeasts to azoles and amphotericin B (AmB), and ET should be used with specific media and longer reading times and only for specific drugs [374].

A completely different approach has been described via corneofungimetry. Stratum corneum cells coated with olive oil form the basement of this testing process, mimicking an in vivo situation [384,385,386]. There is no comparison of this principle with commonly used methods. 

Overall, clear international standard guidelines for susceptibility testing of *Malassezia* are urgently warranted to effectively compare and analyze data, but the authors consider the broth microdilution method the most suitable one and regard this as the gold standard. 

### 10.2. Patterns of Antifungal Susceptibility

For clinical usability, not only the MIC distribution but also other factors such as serum concentration of the drug, pharmacodynamics, resistance mechanisms and clinical efficacy need to be considered [387,388,389]. These are encompassed by clinical breakpoint values established by the CLSI and EUCAST [372,388,389,390]. These breakpoints are regularly updated and if not available, usually the ones for *Candida*, including *C. krusei*, *C. parapsilosis*, *C. tropicalis* and *C. albicans,* are considered [391]. 

Nevertheless, the final proof of resistance is through the detection of the underlying mechanism. For *Malassezia* yeasts, clinical breakpoint values are still not established, but proof of the underlying mechanisms of resistance has been verified for some *Malassezia* species (see below).

Overall, *Malassezia* species antifungal susceptibility profiles against azoles, AmB and terbinafine (TER) vary between species or intraspecies, regardless of the media or other conditions employed [374]. *M. sympodialis* and *M. pachydermatis* are reported to have lower MICs of antifungals AmB, TER and azoles, in general compared to *M. furfur* and *M. globosa* [38,195,378,392,393,394]. 

MIC variation can also be seen within a given species, as shown for *M. sympodialis*, *M. globosa* and *M. furfur* [376,378,393,395]. Similar results are reflected in a canine study involving *M. pachydermatis,* indicating less variation within the same patient but more dissimilarity between different patients [394,396]. 

*Malassezia* spp. bloodstream isolates have higher MICs for the same antifungal drug compared to skin-origin isolates [392,393,397,398,399,400]. Accordingly, the disease status can affect the MIC, as shown in dogs [394,401,402,403]. Patients with prior antifungal exposure showed higher values than healthy individuals. In an in vitro evaluation, strains from diseased dogs showed higher MIC values across several azole drugs, including fluconazole (FCZ), ketoconazole (KZ), miconazole (MIZ), itraconazole (ITZ), voriconazole (VCZ) and posaconazole (PSZ), compared to strains from healthy individuals [404]. Weiler and colleagues found *M. pachydermatis* isolates from diseased animals to be less susceptible to AmB, nystatin, FCZ, clotrimazole (CL) and MIZ [402]. In an Asian study, high MIC values for KZ and ITZ were found among isolates of atopic dogs compared to their healthy counterparts [403].

Not surprisingly, the duration of a disease influences the MIC, as reflected in a canine study on otitis externa, in which patients with chronic disease had higher MIC values associated with MIZ and CL than those with an acute form [394,405]. This could also be related to prior antifungal exposure.

Studies focusing on fungemia have shown a better efficacy of AmB against *M. pachydermatis* than against *M. furfur* [393,395,406]. For *M. furfur*, better effects can be achieved when using the liposomal version of the drug or when combined with FCZ [393,407]. TER works better for *M. pachydermatis* and *M. sympodialis* than for *M. furfur* [395,396,397]. Considering *Malassezia* species overall, ITZ and KZ are reported to be more effective than FCZ, VCZ or AmB [195,392,393,395]. 

Nevertheless, looking at various reports, it can be concluded that for *M. pachydermatis*, ITZ and PSZ show the highest activity compared to other antifungals, with an MIC 90 of mostly less than 0.5 μg/mL. On the other hand, CL (up to 16 μg/mL) and thiabendazole (up to 32 μg/mL) show relatively high values [382,393,396,408,409]. However, from a clinical perspective, MIC 90 (values at which the growth of 90% of the tested isolates is inhibited) warrants careful interpretation since tissue concentrations are not included in the calculation.

## 11. Resistance Mechanisms

Antifungal resistance can be primary (intrinsic) or secondary (acquired) [410]. The former occurs naturally without previous exposure to antifungal drugs. Acquired resistance takes place after or during interactions with antimicrobials [410]. 

An early study in 1994 showed that resistant-induced mutant strains of *M. pachydermatis* exhibited significantly decreased levels of membrane sterols but increased amounts of fecosterol, indicating a possible evasion mechanism of polyene antifungals by replacement of sterol with a precursor product [411]. Mutations in the gene *ERG11* (CYP51), encoding for lanosterol-14α-demethylase, which converts lanosterol to ergosterol, have been detected for induced KZ-resistant *M. pachydermatis* and for clinically resistant *M. globosa* strains. These mutations include missense mutations, amino acid alterations and tandem quadruplication and confer azole resistance [412,413]. Chromosomal rearrangements and gene overexpression, leading to tandem quadruplication of genes within chromosome 4, have been identified in some mutant-resistant strains. Since this region carries genes, including *ERG 4* and *11*, affecting ergosterol synthesis, azole resistance was conferred by this resistance mechanism [413]. 

Overexpression of *ERG11* can also lead to resistance due to the overwhelming presence of the target protein, which has been demonstrated in clinical isolates of *M. pachydermatis* and *M. restricta* [413,414]. 

A different resistance mechanism affecting azole drugs involves efflux pumps. These overexpressed proteins can actively transport accumulated intracellular antifungal drugs out of fungal cells. Around 30 different proteins have been described either belonging to the ATP-binding cassette (such as CDR1, CDR2 or PDR10) or the major facilitator (such as MDR1) superfamily. Such mechanisms have been detected among isolates of *M. pachydermatis*, *M. furfur* and *M. restricta* (Pdr5) [393,414,415,416]. Mitochondrial dysfunction in *M. restricta* strains involving *ATM1*, an iron-sulfur transporter, leading to the activation of the pleiotropic drug resistance (PDR) pathway, resulting in an increased expression of efflux pump transporters, has also been described [414]. Interestingly, by using a *Malassezia* species broth microdilution chequerboard analysis testing the in vitro efficacy of azoles in combination with drug efflux pump modulators (i.e., haloperidol—HAL, promethazine—PTZ, and cyclosporine), FCZ MIC = 128 µg/mL for *M. furfur*, FCZ MIC = 64 µg/mL for *M. pachydermatis* and VOR MIC = 4 µg/mL for both *Malassezia* species were proposed as cut-off values to discriminate suscep2tible and resistant strains [415].

Finally, biofilm formation can also significantly decrease antifungal sensitivity, as shown in studies of *M. pachydermatis* [321,394,409].

## 12. Treatment of *Malassezia*-Related Diseases

### 12.1. Treatment of Malassezia-Associated Skin Diseases

#### 12.1.1. Treatment in Animals

For topical therapy, preparations of chlorhexidine alone or in combination with an azole antifungal are mostly used [2,232,374]. For severe *Malassezia*-associated skin diseases or cases that do not respond to topical therapy alone, oral KZ or ITZ are favored in dogs [2,374,417,418,419,420] and ITZ in cats [2,282,374,421,422]. Due to its high concentration and persistence within the stratum corneum, pulse therapy of ITZ is used with 7 days on, 7 days off, 7 days on, or twice weekly administration [421,423]. Terbinafine [423,424,425,426] and FCZ [427] have been prescribed in single case reports and clinical trials are warranted before treatment recommendations can be made. Even if clinical evidence indicates the efficacy of azole for the control of skin infections, the common recurrence of skin disorders requires the recognition of underlying diseases or the use of prophylaxis systems for the management of these infections in animals [2]. As maintenance therapy, plant-based compounds (i.e., essential oils and phenolic compounds) and peptides have achieved interesting results, but future studies need to be done in order to propose them for clinical use [374].

#### 12.1.2. Treatment in Humans

Pityriasis versicolor—A combination of keratomodulating (sulfur, salicylic acid, selenium sulfide, zinc pyrithione) and antifungal (azoles, ciclopirox olamine, TER) shampoos, sprays or solutions is usually effective, but in widespread, severe, refractory or recurrent cases, systemic antifungal therapy with ITZ or FCZ may be required. Terbinafine is not effective [428,429]. Relapses are common, even after successful initial treatment and long-term management can be challenging [429,430,431] (Figure 3).

Seborrheic dermatitis—Topical treatment with a combination of keratomodulating (pine, tar, salicylic acid, sulfur), antifungal (KZ, ciclopirox, zinc pyrithione) and anti-inflammatory drugs (glucocorticoids, calcineurin inhibitors) together with brushing to remove and soften keratinous material is usually the first choice [219,232,432,433,434,435,436,437]. In severe, widespread and refractory cases, systemic antifungal drugs including ITZ, FCZ, TER and rarely KZ are considered. In addition, it is always important to address the underlying disease if it is present [219,232,432,433,434,435,436,437] (Figure 3).

Malassezia folliculitis—There is some evidence that systemic treatment is the most efficient method, considering the location of the disease within the hair follicles [438]. Itraconazole and FCZ show good efficacy [222,224,439,440,441]. Topical treatment (azoles, selenium sulfide and propylene glycol 50%) may be better used as a preventive measurement or for patients where systemic treatment is contraindicated [439,440,441,442]. Photodynamic therapy as an alternative treatment has also been mentioned [443,444] (Figure 3).

Atopic dermatitis (head and neck dermatitis, HND)—HND patients respond best to systemic antifungal treatment, especially when using ITZ or KZ [243,445,446,447,448,449,450]. Affected individuals are often treated daily for one to two months and then twice weekly for maintenance [448]. Fluconazole can also be used, although some studies report that it would not be as effective as the latter two mentioned drugs [448,451]. Limited data exist for systemic TER [452]. Topical antifungal treatment has not been very promising, although ciclopirox olamine twice daily may be an option for selected cases [453] (Figure 3).

With increased recognition of azole resistance in *Malassezia* species, there has also been an expansion in the investigation of alternative treatment approaches, including photodynamic therapy, natural products, antifungal peptides and proteinase inhibitors [443,454,455,456,457,458,459].

### 12.2. Treatment of Systemic Malassezia Infections

For systemic infections in humans, rapid organism identification, together with an aggressive systemic treatment approach, is essential [14,110,196,460]. Intravenous therapy with AmB is effective in infants and adults [14,16,18,38,72,187,188,191,315,347]. FCZ, PSZ and VCZ have been administered, but careful considerations are necessary since failure of the first two drugs are reported, especially due to reported or suspected reduced susceptibility [18,34,187,188,191,404,461,462,463]. Flucytosine or echinocandins have no efficacy against *Malassezia* and should be avoided [18,185,191,464]. In addition to antifungal therapy, it is of fundamental importance to remove any indwelling devices, such as catheters and to temporarily stop parenteral lipid supplementation [14,18,110,191,196,229,460]. 

## 13. Conclusions

*Malassezia* species are among the most widespread fungi on our planet and it is expected that new species and hosts will be discovered. While some *Malassezia* species are host adapted, many are shared between animals and humans. There is evidence of zoonotic transmission, especially for *M. pachydermatis,* but more longitudinal data are needed for further elucidation. *Malassezia* species can be associated with many different skin diseases in companion, production, avian and exotic animals as well as in humans. In people, *Malassezia* fungemia and internal infections are increasingly recognized, especially in immunocompromised individuals. In addition, these yeasts are associated with certain chronic diseases, such as Crohn’s disease, but also with some cancers, such as pancreatic ductal adenocarcinoma. *Malassezia* species need special culture media to grow and international standardization for susceptibility testing is urgently needed. In both human and veterinary medicine, topical treatment is preferred unless the type, severity or refractory state of the disease doesn’t allow it. For systemic *Malassezia* species infections, AmB is typically used, while for other diseases, azole preparations dominate.

## Figures and Tables

**Table 1:** Classification of *Malassezia* by species, reference strain, hosts and phylogenetic clades.

Species	Reference Strain/GenBank Accession Genome Number	Described Hosts	Clade
*M. furfur*	CBS 14141, GCA_009938135	Human, Cat, Dog, Cattle, Pig, Goat, Elk, Horse, Sheep, Elephant, Monkey, Ostrich, Pelican	A
*M. brasiliensis* *	MA 1455	Parrot	A
*M. yamatoensis*	MY9725, GCA_001264885	Human, Cat	A
*M. psittaci* *	MA 1454	Parrot	A
*M. obtusa*	CBS 7876, GCA_001264985	Human, Cat, Dog, Goat, Horse	A
*M. japonica*	CBS 9431, GCA_001264785	Human, Cat	A
*M. vespertilionis*	CBS 15041, GCA_002818225	Bat	A
*M. globosa*	CBS 7966, GCA_001264805	Human, Cat, Dog, Cattle, Goat, Horse, Sheep, Cheetah	B
*M. restricta*	CBS 7877, GCA_001264765	Human, Cat, Dog, Cattle, Goat, Horse, Sheep	B
*M. arunalokei*	CBS 13387, GCA_020085095	Human, Dog	B
*M. sympodialis*	ATCC 42132, GCA_001264925	Human, Dog, Cat, Pig, Cattle, Goat, Horse, Sheep, Chicken	B
*M. dermatis*	CBS 9169, GCA_001264665	Human, Cat	B
*M. caprae*	CBS 10434, GCA_001264625	Goat, Horse, Human	B
*M. equina*	CBS 9969, GCA_001264685	Horse, Cattle	B
*M. nana*	JCM 12085, GCA_001600835	Cat, Dog, Cattle, Horse	B
*M. pachydermatis*	CBS 1879, GCA_001264975	Human, Dog, Cat, Pig, Goat, Rabbit, Various exotic and wild mammals, Birds (Thraupidae, Macaw)	B
*M. cuniculi*	CBS 11721, GCA_001264635	Rabbit	C
*M. slooffiae*	CBS 7956, GCA_001264965	Human, Cat Cattle, Sheep, Pig, Goat, Horse	C

* = whole genome not available.

**Table 2:** Breed predisposition for *Malassezia* dermatitis in companion animals.

Dog Breeds	Cat Breeds
West Highland White Terrier	Devon Rex
English Setter	Sphynx
Basset Hound	
Boxer	
American Cocker Spaniel	
Poodle	
Dachshund	
Australian Silky Terrier	
Shih Tzu	

## Data Availability

Not applicable.
